# Research progress on human infection with avian influenza H7N9

**DOI:** 10.1007/s11684-020-0739-z

**Published:** 2020-01-23

**Authors:** Xiaoxin Wu, Lanlan Xiao, Lanjuan Li

**Affiliations:** State Key Laboratory for Diagnosis and Treatment of Infectious Diseases, Collaborative Innovation Center for Diagnosis and Treatment of Infectious Diseases, The First Affiliated Hospital, School of Medicine, Zhejiang University, Hangzhou, 310003 China

**Keywords:** H7N9, pandemic, epidemiology, mutations, vaccine, influenza

## Abstract

Since the first case of novel H7N9 infection was reported, China has experienced five epidemics of H7N9. During the fifth wave, a highly pathogenic H7N9 strain emerged. Meanwhile, the H7N9 virus continues to accumulate mutations, and its affinity for the human respiratory epithelial sialic acid 2–6 receptor has increased. Therefore, a pandemic is still possible. In the past 6 years, we have accumulated rich experience in dealing with H7N9, especially in terms of virus tracing, epidemiological research, key site mutation monitoring, critical disease mechanisms, clinical treatment, and vaccine development. In the research fields above, significant progress has been made to effectively control the spread of the epidemic and reduce the fatality rate. To fully document the research progress concerning H7N9, we reviewed the clinical and epidemiological characteristics of H7N9, the key gene mutations of the virus, and H7N9 vaccine, thus providing a scientific basis for further monitoring and prevention of H7N9 influenza epidemics.

**Electronic Supplementary Material:**

Supplementary material is available in the online version of this article at 10.1007/s11684-020-0739-z and is accessible for authorized users.

## Introduction

In March 2013, Shanghai and Anhui were the first two provinces/municipalities to report human cases of avian influenza H7N9 infection. Since then, the disease has spread, and cases of H7N9 have increased rapidly in Zhejiang Province. To date, five waves of H7N9 outbreaks have occurred, and H7N9 cases have spread to most provinces and cities in China, among which Zhejiang, Jiangsu, and Guangdong are the most commonly affected provinces [1]. In addition to China, cases of H7N9 have also been reported abroad [2,3]. By tracing the source of the virus, the H7N9 virus was found to originate from the live poultry market, and its homology with poultry virus was as high as 99.4%. The H7N9 virus has eight gene fragments, including H7 and N9 gene, and six internal protein genes (polymerase basic protein 1 (PB1), polymerase basic protein 2 (PB2), nucleocapsid protein (NP), polymerase acidic protein (PA), matrix protein (M), and non-structural protein (NS)). It is a new reassortment virus. The NA gene is from H7N9, the HA gene is from H7N3, and the six internal genes of the isolate H7N9 are closest to those of poultry H9N2 viruses of China [4]. The new H7N9 virus shows low pathogenicity to birds, which do not show severe symptoms or die after infection. However, when it infects humans, it can lead to severe symptoms and even death. From 2013 to 2017, five waves of H7N9 outbreaks have occurred, with a case fatality rate of approximately 40% [5].

The H7N9 virus evolved and mutated over many years. In 2017, a new highly pathogenic H7N9 virus emerged and infected humans, quickly spreading to eight provinces and cities in China. This new H7N9 virus had four amino acids inserted into the HA gene splice site compared with previous H7N9 viruses [6,7]. Through tracing analysis, this H7N9 virus appeared to originate from the Yangtze River Delta, and this gene mutation was obtained in the Pearl River Delta region, where it recombined with low-pathogenic H7N9 and H9N2 from local poultry sources to become a new type of highly pathogenic H7N9 virus [7,8]. The mutation occurred during the fifth wave of H7N9. The fifth wave involved the largest number of cases and was the most serious outbreak since 2013 [5,9]. Meanwhile, the H7N9 virus continues to accumulate mutations, and its affinity for human respiratory epithelial sialic acid 2-6 receptor is increasing. Therefore, a pandemic is still possible [10,11]. This article reviews the clinical and epidemiological characteristics of H7N9 avian influenza, the key gene mutations of the virus, and H7N9 vaccines. It also provides a scientific basis for further monitoring and prevention of H7N9 influenza epidemics.

## Clinical characteristics of H7N9 avian influenza virus

### Clinical symptoms

Patients infected with H7N9 generally show symptoms of pneumonia, often presenting as fever, cough, and expectoration, accompanied by muscle pain, headache, chest tightness, diarrhea, vomiting, and general malaise. In the case of fever, the body temperature persists above 39 °C, and breathing is accelerated. In a case of severe disease, the disease progresses rapidly. Acute respiratory distress syndrome (ARDS), septic shock, multi-organ failure, and even death will occur within 3–7 days [12]. In a few patients, the infection can be mild, only presenting as fever with upper respiratory tract infection [13,14].

### Laboratory examination

Routine blood examination showed that the total number of early white blood cells is generally not high or low. Severe cases exhibit lymphocytopenia and thrombocytopenia. Blood biochemical examination may include C-reactive protein, alanine aminotransferase, aspartate aminotransferase, lactate dehydrogenase, creatine kinase, and myoglobin elevation [12,14,15].

### Etiology and related detection

Respiratory tract specimens, including nasopharyngeal secretions, sputum, airway aspirates, and bronchoalveolar lavage fluid, are mostly used for etiological detection. The positive rate of lower respiratory tract specimen is higher than that of upper respiratory tract specimen. Samples must be submitted for inspection in time after retention. Nucleic acid detection of H7N9 virus is the most commonly used and convenient and rapid method of pathogen detection. Nucleic acid detection should be the first choice for suspected human infection with H7N9 avian influenza [16]. Nucleic acids in respiratory secretions should be tested regularly in all positive cases until they become negative.

Isolation of the H7N9 virus is another way to confirm the diagnosis. The isolated H7N9 strain can be retained, providing valuable materials for follow-up treatment and research. To isolate H7N9 avian influenza virus from respiratory specimens of patients, the quality of respiratory samples must be ensured. Generally, viruses can be isolated if the cycle threshold value of quantitative PCR from respiratory samples is below 28. Madin–Darby canine kidney (MDCK) cells and chicken embryos are the most commonly used vectors for H7N9 virus isolation. When viruses are isolated using MDCK cells, additional L-1-tosylamide-2-phenylethyl chloromethyl ketone (TPCK)-treated trypsin at a final concentration of 2 μg/mL is required to assist virus replication. There is no need to add TPCK trypsin to isolate H7N9 virus using chicken embryos. Filtration and sterilization of respiratory tract samples can improve the virus isolation rate. MDCK cells are mammal-derived cell lines; therefore, the probability of virus mutation is low when viruses are isolated; however, the yield of virus isolated by MDCK cells is small. H7N9 virus may mutate during isolation using chicken embryo because they are poultry cells; however, the yield of virus is high after isolation from chicken embryos. Currently, both methods are often used [16]. In addition, respiratory samples can be stored for up to one month when frozen at −80 °C after the addition of virus preservative.

Serological detection can also be used to determine H7N9 infection. Levels of antibodies specific for H7N9 avian influenza virus in the serum at the acute and convalescent stages can be determined using dynamic detection. Serum antibody levels in the convalescent stage are four or more times higher than those in the acute stage, which is generally not used for diagnosis [14,15]. The immunocolloid gold technique can also be used to diagnose H7N9 virus. However, compared with the quantitative PCR method, the sensitivity of immunocolloid gold technique is poor, but its specificity is good, reaching 97.56%. Compared with virus culture method, the sensitivity and specificity of immunocolloid gold technique are better, making it useful as a rapid screening method with a certain clinical diagnostic value [16].

In addition, some studies have found that in severe and mild cases, H7N9 virus nucleic acid can be detected in the feces of patients, showing a positive rate of 67% and 33%, respectively. Feces can also be used as an auxiliary sample source for H7N9 virus infection [17]. In a study involving 14 patients infected with H7N9 patients, Hu *et al.* found that H7N9 virus nucleic acids could be detected in the patient's serum, feces, and urine, suggesting that these samples can also be used as sources of H7N9 virus nucleic acids and have potential significance in clinical detection [18].

### Imaging examination

X-ray and computed tomography (CT) are most commonly used for imaging examination. Patients infected with H7N9 develop pneumonia, and their lungs exhibit patchy shadows. Severe lesions progress rapidly and exhibit double lung grinding glass shadows, and lung consolidation images can be accompanied by a small amount of pleural effusion. In the case of ARDS, the lesions are widely distributed [19,20]. Ultrasound is also of certain significance in the examination of H7N9 patients and can be used as auxiliary examination or temporary replacement examination [21].

## Pathological examination

The lung often presents with acute exudative inflammatory changes, pulmonary hemorrhage, diffuse alveolar injury, and transparent membrane formation. In kidney and liver tissues, ischemic and fatty lesions may be present. Splenic lymphocyte atrophy may occur in the spleen. Reactive hemophagocytosis can be found in the bone marrow [17,20].

## Diagnosis

Epidemiological investigation can be used to indicate the diagnosis of H7N9 virus infection. Generally, within 2 weeks before the onset of the disease, patients slaughter live birds, come in contact with birds and their excreta and secretions, go to live poultry markets [22], or have close contact with human cases of H7N9 avian influenza. The main source of human infection with H7N9 is believed to be live poultry. Limited human-to-human transmission exists sporadically, and no evidence of multigenerational transmission currently exists [23]. In addition to the epidemiological history and clinical symptoms, the only way to diagnose H7N9 virus infection is to detect the H7N9 pathogen. H7N9 infections can be classified as mild or severe. Mild infections present with upper respiratory symptoms. The confirmation of severe cases needs to meet the following conditions, which require mechanical ventilation with endotracheal intubation or vasoactive drug therapy after active fluid resuscitation for septic shock. Severe cases can be diagnosed in three of the following six conditions: respiratory rate 30 times/min, oxygenation index lower than 250 mmHg, disturbance of consciousness and/or disorientation, multiple lobar infiltration, blood urea nitrogen higher than 7.14 mmol/L, or systolic blood pressure $< 90$ mmHg requiring active fluid resuscitation. The main risk factors for severe disease are age over 65 years; smoking history; complicated with severe basic diseases or special clinical conditions, such as heart or basic lung diseases, hypertension, diabetes, liver disease, obesity, and tumors; immunosuppression; and pregnant and parturient women. After onset, the fever is continuously high ( $> 39$ °C); the lymphocyte count continuously decreases; C-reactive protein, lactate dehydrogenase, and creatine kinase continuously increase; and chest imaging indicates rapid progress of pneumonia [14,24].

## Treatment

Confirmed and suspected cases should be isolated as soon as possible. The most basic symptomatic treatment includes oxygen therapy, cooling, cough relief, and dispelling phlegm. According to the degree of hypoxia, a nasal catheter, nasal high-flow oxygen therapy, open mask, and oxygen storage mask can be used for oxygen therapy. High fever can be physically cooled or treated with antipyretic drugs. Patients with severe cough and phlegm may be given cough expectorant drugs [14]. Four-anti and two-balance strategies should be followed in clinical treatment (Fig. 1). The so-called four-anti and two-balance strategies include antiviral, anti-shock, anti-hypoxemia and anti-multi-organ failure, and anti-secondary infection strategies and maintaining water–electrolyte acid–base balance and maintaining the microecological balance [12].

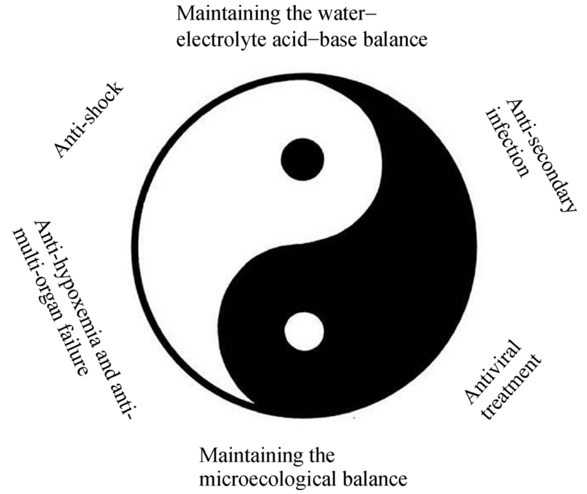

**Fig. 1** Schematic of the four-anti and two-balance strategies used in treating severe H7N9. The balance between Yin and Yang in the human body is very important. The four-anti and two-balance strategies are used to maintain this balance. Each strategy (antiviral treatment, anti-shock, anti-hypoxemia and anti-multi-organ failure, anti-secondary infection, maintaining the water–electrolyte acid – base balance, and maintaining the microecological balance) plays an important role in the treatment of severe H7N9.

### *Antiviral treatment*

Currently, the antiviral drugs used to treat patients with H7N9 mainly include neuraminidase (NA) inhibitors. These drugs should be used early for suspected and confirmed cases. NA inhibitors are recommended within 48 h. The mortality of patients treated with NA inhibitors within 48 h is significantly lower than that of patients treated within 2–5 days and 5 days after diagnosis. The earlier the treatment, the better. After treatment with NA inhibitors within 48 h, patients generally become negative for the virus after 3–9 days (median 4.5 days) [25]. The commonly used NA inhibitors include oseltamivir, peramivir, and zanamivir. Oseltamivir is given to adults at 75 mg twice daily until the virus becomes negative. The dose for severe cases can be doubled, and the course of treatment can be extended appropriately. Children aged 1 year and above should be given medicine according to their weight (a child dosage form should be selected). Peramivir can be used in severe cases or in individuals who cannot take it orally. The adult dosage is 300–600 mg once a day via intravenous drip until the virus turns negative. The dosage can be adjusted according to clinical needs. Zanamivir is available for ages 7 years and older. The dosage is 10 mg (inhalation) twice a day and 12 h apart. Zanamivir is currently used sparsely and is not recommended for patients with severe illness or complications [14]. According to the specific condition of patients, a single drug or combination of drugs can be used empirically. In a retrospective study of H7N9 treatment, Zhang *et al*. found that oseltamivir combined with peramivir or oseltamivir alone had no effect on the treatment effect of H7N9 patients. In this study, 43 patients treated with monotherapy and 39 patients treated with combination therapy were included, and no statistical differences in the probability and mortality of ARDS was observed between the two groups [26]. Before medication, patients should be collected for virus isolation, drug sensitivity testing, and drug selection and dose adjustment according to their drug sensitivity. There have been experimental reports of drug resistance changes in H7N9. Therefore, in addition to existing drugs, new drugs still need to be developed.

Traditional Chinese medicine also plays an important role in the treatment of H7N9 patients [27]. Luo *et al.* showed that honeysuckle and forsythia can effectively prevent and control H7N9 influenza virus through a synergistic molecular effect. Ethyl linoleate and forsythoside A could be further developed as potential inhibitors of H7N9 influenza virus [28]. Thermonin combined with NA inhibitors also plays a role in the treatment of H7N9 and has achieved good clinical effects [14].

### *Anti-shock*

Maintaining effective perfusion of body organs and protecting their functions is important. This strategy includes dynamic hemodynamic detection, sufficient and effective fluid resuscitation, and reasonable selection of vasoactive drugs [15]. When a cytokine storm is present, Li’s artificial liver system should be used in time to treat and reduce the condition. Three hours after plasma exchange combined with continuous hemofiltration, the cytokines and chemokines of patients with H7N9 were found to be significantly reduced and maintained at a low level [29].

### *Anti-hypoxemia and anti-multi-organ failure*

H7N9 avian influenza virus mainly affects the respiratory system; therefore, respiratory failure is the main cause of death of patients. Major measures to correct hypoxemia include oxygen therapy, mechanical ventilation, and extracorporeal membrane oxygenation (ECMO). Oxygen therapy and mechanical ventilation are most commonly used to support respiratory function. After 2 h of oxygen therapy, if the peripheral capillary oxygen saturation ($SpO_2$) is still less than 92% or if the patient still shows dyspnea and the improvement of respiratory distress is not obvious, mechanical ventilation should be conducted. Non-invasive ventilation can be attempted at an early stage, and an oral and nasal mask is recommended. The treatment of ARDS can be conducted according to the principle of mechanical ventilation. Complications such as mediastinal emphysema and ventilator-associated pneumonia may occur in the treatment of ARDS, which should be noted. If non-invasive ventilation treatment for 1 to 2 h does not improve the condition, clinicians may need to consider the implementation of invasive ventilation as soon as possible. When ARDS protective ventilation strategy is applied, the negative expiratory pressure tidal volume is adopted, and appropriate positive end-expiratory pressure is selected to achieve active lung re-expansion; prone ventilation is adopted in severe cases [14]. ECMO can be selected according to the conditions of patients. ECMO can effectively improve oxygenation and plays an important role in rescuing patients with severe H7N9 infection [18,30–37]. Renal replacement therapy and artificial liver therapy may be used in patients with impending multiple organ dysfunction.

### *Anti-secondary infection*

Patients with H7N9 are prone to secondary infection during long-term intensive care unit treatment. Clinical manifestations and laboratory data can be used to determine whether or not bacterial infection is complicated and whether there is sufficient evidence suggesting that the secondary habitual infection may be treated with antibiotics [38]. Specimens need to be cultured before using antimicrobial agents to find the source of infection.

### *Maintaining the water–electrolyte acid–base balance*

The water–electrolyte acid–base balance plays a key role in maintaining normal life activities. Patients with H7N9, especially severe cases, are prone to water–electrolyte acid–base disorder. Maintaining the water–electrolyte acid–base balance is very significant in clinical treatment. During treatment, water and electrolyte levels, blood osmotic pressure, and arterial blood gas should be monitored regularly. At the same time, fluid intake should be maintained: 50 mL/kg weight daily fluid is generally recommended, which may increase or decrease according to the specific clinical situation. Obvious pulmonary exudation in patients with H7N9 indicates that a negative balance can be properly maintained to alleviate pulmonary exudation and improve oxygenation. Li's artificial liver treatment system can also play an important role in correcting water and electrolyte disorders [29].

*Maintaining the microecological balance*

Normal intestinal flora play an important role in the body; they maintain the intestinal barrier, regulate immune function, provide colonization resistance, lower blood ammonia and cholesterol, promote nutrition, and exert anti-aging effects. Using second-generation sequencing technology, Qin *et al*. found that H7N9 virus infection and antibiotic administration had a significant impact on the host's microbial community, leading to a decline in the diversity and overgrowth of bacteria such as *Escherichia coli* and *Enterococcus faecalis*. The use of microecological agents can improve the diversity of intestinal microorganisms and the richness of beneficial bacteria [39]. Hu *et al*. found that intestinal flora diversity and population richness of patients with H7N9 were reduced significantly. Butyric acid did not reduce or improve secondary infections. *Bacillus subtilis* and *Enterococcus faecalis* may play a role in reducing or improving secondary infection in these patients [40]. Lu *et al*. found that the ratio of *Bifidobacteria* to Enterobacteriaceae in patients with H7N9 patients was less than 1. Most healthy volunteers had a ratio greater than 1. Elderly patients with H7N9 are prone to secondary bacterial infection, and the repair and treatment of their microecology can significantly reduce enterogenous secondary bacterial infections [41].

### Intensification mechanism of H7N9 patients

Studies have found that patients with H7N9 avian influenza often do not have high levels of inflammatory cytokines, whereas those with severe cases have obvious cytokine storms [42]. When H7N9 infects humans, it can trigger cytokine storms that can lead to systemic inflammation, respiratory distress syndrome, shock, multi-organ failure, and death. Among the 48 cytokines and chemokines measured, 34 cytokines and chemokines were significantly increased. Among them, the increase of interferon-induced protein 10 (IP-10), which is related to lung injury, was the most obvious. Cytokines such as macrophage migration inhibitory factor (MIF), stem cell factor (SCF), hepatocyte growth factor (HGF), monocyte chemokine protein 1, stem cell growth factor (SCGF), IP-10, interleukin (IL)-18, and interferon are closely related to H7N9 severity and can effectively predict patient prognosis. IP-10, interferon γ-induced mononuclear cytokines, MIF, HGF, IL-18, SCF, nerve growth factor, and SCGF levels are positively correlated with H7N9 viral load [43]. In a previous study, angiotensin II was found to be significantly increased in patients with H7N9 and was significantly higher than that in H1N1 patients and the normal control group; it was positively correlated with viral load in patients with H7N9; thus, angiotensin II could indicate patient prognosis [44].

## Epidemiological characteristics of H7N9 avian influenza

### Epidemic season

Human infection with H7N9 bird flu has obvious seasonal distribution characteristics. Generally, it comes during winter and spring and peaks in December to April, with the highest incidence in January and February [5].

### Source of infection

Birds carrying H7N9 avian influenza virus or an environment contaminated by the virus are the main sources of H7N9 infection [4]. To date, several cases of familial clusters have been observed, but most of them are sporadic. Moreover, no evidence of sustained human-to-human transmission has been documented. Patients with H7N9 can also serve as a source of infection. They should be isolated and treated to avoid nosocomial infection [23,45]. The H7N9 virus has also been detected in poultry eggs, representing a potential source of infection; however, no relevant evidence of such transmission has been noted [45]. Closing a live poultry market can effectively block the transmission of H7N9 virus from birds to humans [22]. However, shutting down all live poultry markets nationwide, even in areas with high incidence of H7N9, is unrealistic. Intermittent closure of live poultry markets, or closure of live poultry markets at night, can effectively reduce the source of the virus by separating aquatic birds, such as ducks and geese, from those that live on land [46].

### Transmission route

Human infection with avian influenza H7N9 occurs mainly through respiratory tract transmission, from close contact with infected bird feces or secretions, or through exposure to virus-contaminated environments.

### Susceptible people

People without antibodies to H7N9 are at risk of infection. Currently, people infected with H7N9 avian influenza are mainly middle-aged and elderly [47]. The infection rate in rural areas is higher than that in urban areas [48].

## Key genetic variations of H7N9 virus

The key genetic variations of H7N9 virus are shown in Table 1.

### Hemagglutinin (HA)

HA contains two subunits, HA1 and HA2. HA plays a key role in the invasion of influenza virus into host cells. HA can bind to the sialic acid receptor of host cells, thus initiating cell invasion. Avian influenza viruses mainly bind to 2-3 sialic acid receptors, whereas human influenza viruses mainly bind to 2-6 sialic acid receptors. Human upper respiratory tract cells mainly express 2-6 sialic acid receptors. The HA gene is highly variable; therefore, it is prone to mutation, leading to changes in the affinity between HA and sialic acid receptors. When the HA gene changes at the Q226L and G228S loci, it increases its affinity for the sialic acid 2-6 receptor, leading to increased human susceptibility [49]. Mutations at G186V also enhance the virus' affinity for sialic acid 2-6 receptors. These sites were found in abundance early in H2N2 and H3N2. The G228S mutation has been identified in the H7N9 virus [50]. Changes in Q226L and G186V have also been found in H7N9 viruses, making the H7N9 virus more susceptible to infection from birds to humans [51–53]. In a study conducted in Guizhou Province, mutations at G186V and Q226L were also found in HA receptor sites of the strains from 2014 to 2017 [54]. In a study in Jiangsu Province, 41 among 167 H7N9 strains showed G186V and Q226L/I changes in HA sites [55], and H7N9 strains from Hangzhou also showed changes in the Q226I site [56]. Mutations at the G186V and Q226L loci have also been found in H7N9 virus isolated from terrestrial birds, leading to an increased possibility of human infection [57].

When the HA splicing site was inserted into the four amino acids of KRTA, low-pathogenic H7N9 became a highly pathogenic H7N9 [6,7]. HA is also the main antigenic component of influenza and is an important target protein of neutralizing antibodies. When HA changes, the original immunity decreases or the vaccine becomes ineffective. Some studies have found that when A143V and R148K emerged on the HA protein of some H7N9 strains, neutralization of the original antibodies was significantly reduced, leading to immune escape of the virus [58]. A ferret experiment showed that when H7N9 comprised the L217Q mutation, the antigenicity was significantly changed, resulting in a marked decrease in the titer of the original antibody. Monitoring the change of this site is of great significance for vaccine development and upgrading [59].

**Table 1** Key genetic variations of the H7N9 virus

| Gene | Function | Mutation | References |
|---|---|---|---|
| HA[a] | Change of antigenicity | A143V | [58] |
| | Change of antigenicity | R148K | [58] |
| | Change of antigenicity | L217Q | [59] |
| | Increases affinity for sialic acid α2-6 receptor | Q226L/I | [49, 51–57] |
| | Increases affinity for sialic acid α2-6 receptor | G186V | [51–55, 57] |
| | Increases affinity for sialic acid α2-6 receptor | G228S | [50] |
| | Cleavage peptides | PEVPKRKRTAR↓G | [6, 7] |
| NA[b] | Reduces drug sensitivity | R292K | [54, 60, 61] |
| | Reduces drug sensitivity | H274Y | [61] |
| | Reduces drug sensitivity | E119V | [62] |
| | Reduces drug sensitivity | I222K | [63] |
| PB2 | Enhances viral transcription and replication in cells | K526R | [61] |
| | Enhances viral transcription and replication in cells | E627K | [51, 64, 65] |
| | Increases virulence in mammalian models | D701N | [51, 64, 65] |
| | Restores polymerase activity | M535L | [66–68] |
| | Host signature amino acids (avian to human) | T271A | [69] |
| | Increases viral replication and virulence | Q591K | [66–68] |
| | Increases viral replication and virulence | A588V | [8] |
| | Host signature amino acids (avian to human) | K702R | [7, 70] |

[a]The H3 numbering system was used. [b]The N2 numbering system was used.

### NA

NA has the function of hydrolyzing sialic acid. When influenza virus matures, it binds to host cell sialic acid receptors through HA, which requires NA to separate the virus from the host cell surface. Therefore, NA has become an important target for anti-influenza drugs. Mutations in NA are likely to result in reduced drug sensitivity or drug resistance to existing NA inhibitors. Mutations at the R292K and H274Y (N2 numbering) sites promote oseltamivir and peramivir resistance, and these sites have been reported in seasonal influenza H1N1 and H1N1-pdm2009. Wan *et al.* found that some human infections of H7N9 virus strains have mutations at the resistance site of R292K (N2 numbering) [54]. Zou *et al.* included 11 patients with H7N9 and isolated the R292K (N2) site related to oseltamivir resistance from specimens of five patients [60]. Dong *et al.* found that some H7N9 strains had mutations in both R292K and H274Y (N2 numbering) [61]. E119V is also an important resistance site, which is often found in H3N2 viruses. Marjuki *et al.* also found a mutation at the E119V site in viruses isolated from patients with H7N9 in Taiwan, which also resulted in decreased sensitivity to oseltamivir. In addition, oseltamivir sensitivity also decreased when I222K was present, which was confirmed in ferrets [62,63].

### Polymerase basic protein 2 (PB2)

In the early separation of human infection with H7N9 strains, existing D701N and E627K mutations, which allow H7N9 to more easily replicate in mammals, were identified [51]. Another study found that in addition to D701N and E627K, the mutation A588V could also increase viral replication in mammals, making the H7N9 virus more pathogenic to humans [8]. Meanwhile, a study in the Suzhou area found that the H7N9 virus could have both K526R and E627K mutations, which makes the virus more likely to replicate [61]. By monitoring poultry samples, changes in these key sites were observed, and H7N9 has acquired the mutation of E627K or D701N in poultry, which increases the risk of human H7N9 infection from birds [64,65]. In addition, other studies have found that an increasing number of H7N9 strains possess the two mutations of M535L and Q591K, which potentially enhance the possibility of human infection and may increase the virulence of H7N9 [66–68]. Meanwhile, studies have found that T271A, Q591K, and D701N can complement each other in increasing infectivity [69]. K702R is an important mutation in the avian influenza virus site, which makes the virus more infective to humans. Some studies have found that certain H7N9 strains have mutations at this site, increasing their infectivity to humans [7,70].

## Research status of H7N9 vaccine

Avian influenza A H7N9 virus is a new kind of virus. Its antigenic characteristics are different from those of previous influenza viruses. Specific vaccines for H7N9 are under development and have been submitted for clinical trials; however, no vaccine has been approved for sale. The World Health Organization has announced several vaccine candidates for H7N9. The vaccine candidates were from several basic strains, including A/Anhui/1/13 (H7N9), A/Shanghai/2/2013(H7N9), A/Hongkong/125/2017(H7N9), and A/Guangdong/17sf003/2016 (H7N9). Currently, the vaccine seeds that are involved in clinical trials are mainly from two strains, A/Anhui/1/13 (H7N9) and A/Shanghai/2/2013(H7N9).

Since the H7N9 outbreak in 2013, a vaccine has been put into clinical trials. This vaccine is a virus-like particle vaccine from A/Anhui/1/13(H7N9). A recombinant baculovirus was expressed in insect cells comprising the HA and NA genes derived from A/Anhui/1/13(H7N9) and M1 from A/Indonesia/5/05. The trials used a new adjuvant, ISCOMATRIX. This adjuvant is developed on the basis of ISCOMS (spherical cage particles composed of saponins, cholesterol, phospholipids, and antigens), which is a brand new antigen presentation system with dual functions of adjuvant and antigen presentation. In essence, it has the same composition and structure as ISCOMS but also comprises cholesterol, phospholipids, and saponins without antigens. The clinical trial enrolled 284 adults who were immunized with a two-injection regimen for 0 to 21 days and monitored for side effects and safety. The experiment was divided into seven groups: a placebo group, 15 and 45 μg groups, 5 and 15 μg plus 30 units of adjuvant groups, and 5 and 15 μg plus 60 units of adjuvant groups. The results showed that 60 units of ISCOMATRIX had the best effect in the 5 μg group. Two weeks after immunization, the titer of hemagglutination inhibition reached 64.3 (95% confidence interval, 485–85.3), and the serum positive conversion rate reached 80.6% (64.0%–91.8%). However, the addition of ISCOMATRIX adjuvant increased local and systemic adverse events and may easily lead to the recurrence of previous diseases [71].

In 2014, a vaccine derived from MDCK cell culture was also put into clinical trials. The HA and NA proteins of this vaccine were derived from A/Shanghai/2/2013(H7N9), which was recombined with PR8 through reverse genetics and cultured in MDCK cells. The MF59 adjuvant was used in this clinical trial. The phase I trial recruited 402 subjects. Results showed that 15 μg HA plus 0.25 mL of MF59 adjuvant had the best immune effect. The positive conversion rate of the hemagglutination inhibitory antibody and the microneutralizing antibody reached 78% at 3 weeks after injection, with no significant increase in side effects [72].

From September 2013 to May 2014, a pasteurization-derived vaccine based on A/Shanghai/2/13 (H7N9) was used in a US multi-center phase II clinical trial. The clinical trial recruited 700 subjects between the ages of 19 and 64 years. The 3.75 μg vaccine plus MF59 adjuvant had the best effect in both the first dose and the booster immunization. The titer of serum neutralizing antibodies reached 81.4, and the positive rate of antibody reached 81%. The results also showed that the H7N9 vaccine was more effective than the previous flu vaccine. The younger the person, the better the immune response. The experiment also found that the use of MF59 adjuvant increased the local reaction in the arm [73].

From September 2013 to January 2015, a monovalent inactivated vaccine based on A/Shanghai/2/2013(H7N9) strain (produced in the same way as the seasonal flu vaccine) was also conducted in the USA for a phase II clinical trial. This trial recruited 980 people, aged 19–64 years, who were also immunized over 0–21 days using the two-injection method. The tests were divided into 3.75, 7.5, 15, and 45 μg of HA, containing either AS03 (tocopherol and squalene aqueous emulsion) or MF59. The results showed that the antibody level in response to the 3.75 μg plus AS03 dose was the best, and the titer of neutralizing antibodies reached 211.9 at 8 days after the two immunizations. The titer of neutralizing antibodies remained at 170.9 at 3 weeks after immunization. This suggested that the AS03 adjuvant might be superior to the MF59 adjuvant in combination with an H7N9 vaccine. At the same time, the antibody levels of each group at all ages were compared 21 days after the second immunization, and the immune effect was good in patients aged 19–34 years old, suggesting that the vaccine had a better effect on young people [74].

Another trial, conducted in Canada and the USA, involved 424 and 386 participants in phase I and phase II clinical trials, respectively. The experimental vaccine was based on A/Shanghai/2/2013 (H7N9), an inactivated vaccine produced using reverse genetics. The adjuvants were 5.93 mg AS03B or 11.86 mg AS03A. The clinical trial was followed for 385 days. The results showed that 11.86 mg AS03A combined with 7.5 μg of vaccine had the best protective effect against H7N9 after immunization, and the titer of hemagglutination inhibitory antibodies reached 151.1 at 21 days after the two injections [75]. Thus, AS03 could significantly increase the antibody level in response to the H7N9 vaccine.

A recent report on a live attenuated vaccine was published. Forty subjects were recruited for this clinical trial. The experimental and control groups were constructed at a ratio of 3:1. The HA and NA genes of this recombinant virus were from A/Anhui/1/2013 (H7N9), and the remaining six gene fragments were from the Leningrad/134/17/57 (H2N2) cold adaptation virus. The experimental results showed that the attenuated live vaccine did not lead to a significant increase in side effects [76].

In 2017, a team composed of people in Taiwan performed clinical trials of a vaccine derived from MDCK cells. The seed strains of the vaccine were based on the Anhui strain. This experiment used the vaccine after the culture and lysis of MDCK cells, with 40 people in the first phase and 160 people in the second phase. Approximately 15 or 30 μg of vaccine, with or without an aluminum adjuvant, was used. The overall effect of the vaccine was poor, and the levels of the hemagglutination inhibitory antibodies and neutralizing antibodies did not reach 40. In these four groups of tests, the effect of 30 μg plus aluminum adjuvant was relatively good [77].

Currently, clinical trials of H7N9 vaccines are still ongoing. Some clinical trials have been completed and new ones are still under way (Supplementary Tables S1 and S2). The USA, Australia, and other countries are leading clinical trials of H7N9 vaccines. Clinical trials have been carried out in different countries with different strains of vaccine, different types of vaccine (lysed vaccine or live attenuated vaccine), and different doses of vaccine mixed with different adjuvants. Currently, China's H7N9 vaccine research mainly uses Anhui and Zhejiang strains. The seed strain of H7N9 vaccines is constructed by reverse genetic method (Fig. 2). Clinical trials of H7N9 vaccines are underway in China, and phase I and II trials have been completed in Taiwan. Phase II clinical trials have been conducted in Chinese mainland. The lack of intellectual property rights and experience in the use of MF59, AS03, ISCOMATRIX, and other adjuvants indicates that the adjuvants currently used in clinical trials in Chinese mainland are mainly based on aluminum adjuvants. With the broadening of clinical trials, the clinical data for H7N9 vaccines will be constantly improved, laying a solid foundation for the storage and marketing of H7N9 vaccines.

## Conclusions

H7N9 is an avian influenza virus that is harmful to human beings. In the past 6 years, H7N9 infections have continuously occurred, and the virus has accumulated mutations, increasing its adaptability and drug resistance in humans. Meanwhile, a new type of highly pathogenic H7N9 bird flu emerged in 2017, spreading to eight provinces/municipalities in a short time. Although the fatality rate of highly pathogenic H7N9 avian influenza and previous H7N9 avian influenza is not significantly different, highly pathogenic H7N9 avian influenza has a large number of acquired adaptive mutation and drug resistance sites within a short period of time. Thus, the spread of this highly pathogenic H7N9 avian influenza still requires attention. Meanwhile, the H7N9 virus continues to

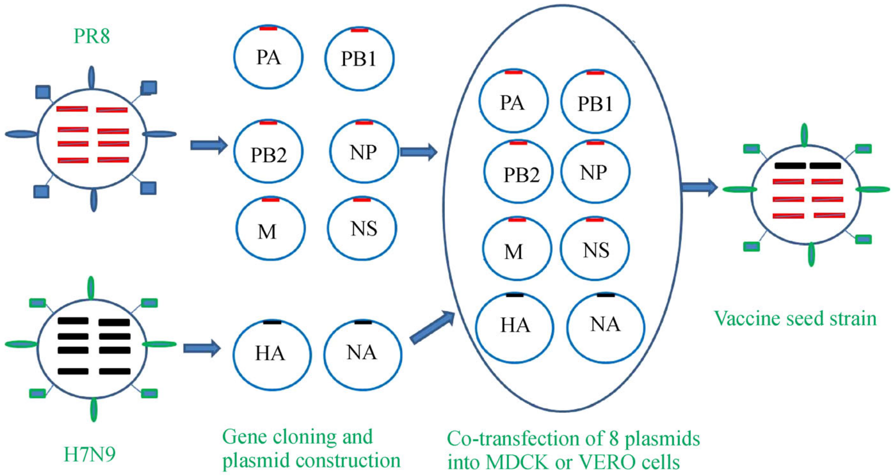

**Fig. 2** Seed strain construction of H7N9 vaccine by reverse genetic method. The HA and NA genes were from the H7N9 strain. All other internal genes (PB1, PB2, NS, M, NP, PA) were derived from A/Puerto Rico/8/34 (PR8). All the genes were constructed into pHW2000 plasmid. Then, all eight plasmids were co-transfected into MDCK or VERO cells. The seed strain of H7N9 vaccine could be collected in culture supernatant.

be isolated from both environmental and poultry samples, indicating that the H7N9 virus can re-infect humans and cause a new wave of outbreaks.

At present, H7N9 infection in humans has been brought under control by shutting down live poultry markets and injecting H5 and H7 vaccines into poultry in many provinces/municipalities across the country. At present, no sixth H7N9 epidemic has occurred [78]. Although domestic and foreign academic researchers have made remarkable progress in the research into H7N9 within a short time, many problems remain to be solved. The fatality caused by H7N9 has been reduced to less than 10% in some areas; however, it is still as high as 40% nationwide. Reducing the death rate nationwide remains a huge challenge. A small number of patients do not have a clear history of poultry or viral exposure, and the source of their infection cannot be explained. The H7N9 virus can be transmitted from person to person with limited frequency. However, its mechanism and ability to evolve into a strain with more frequent human-to-human transmission remain unknown. The H7N9 virus mainly infects middle-aged and elderly people. Workers with a history of contact with H7N9 virus are not necessarily infected with this virus. The selective infection mechanism of H7N9 in the population is still unknown. Currently, poultry is under effective supervision; however, wild waterfowl and migratory birds may still become hosts of H7N9, which are mostly out of human control. H7N9 vaccines for humans are currently undergoing clinical trials. However, none are available on the market. Thus, the risk of infection is still present.

Considering the high mortality of H7N9 in China, national educational workshops should be conducted to popularize the four-anti and two-balance strategies of severe H7N9 treatment, to set up teams that can prevent and treat H7N9 in all parts of the country, and to popularize the experience of ECMO and artificial liver system in order to decrease the fatality rate of H7N9 in the future. The selective infection mechanism of H7N9 in the population should be studied. In our experience, four main factors determine whether people will be infected with H7N9, namely, virus load, receptor quantity, receptor affinity, and host immunity. We should focus on studies of receptor affinity and the interaction between virus and host. At the same time, we should control the source of H7N9 virus and monitor and control poultry, wild birds, and migratory birds. At present, poultry has been controlled; thus, we should strengthen the monitoring and inspection of wild birds and migratory birds and take timely and effective measures to prevent the spread of the virus. Monitoring and control of H7N9 are still a long way off. Further clinical trials of the H7N9 vaccine still need to be conducted. Continuously strengthening and deepening research into H7N9 will make it possible for us to detect and track variations and to prepare for outbreaks.

## Acknowledgements

This work was supported by the State Project of Essential Drug Research and Development (No. 2015ZX09101044).

## Compliance with ethics guidelines

Xiaoxin Wu, Lanlan Xiao, and Lanjuan Li declare that they have no financial conflicts of interest. This manuscript is a review article and does not require approval by a relevant institutional review board or ethics committee.

**Electronic Supplementary Material** Supplementary material is available in the online version of this article at https://doi.org/10.1007/s11684-020-0739-z and is accessible for authorized users.

**Open Access** This article is licensed under a Creative Commons Attribution 4.0 International License, which permits use, sharing, adaptation, distribution and reproduction in any medium or format, as long as you give appropriate credit to the original author(s) and the source, provide a link to the Creative Commons licence, and indicate if changes were made.

The images or other third party material in this article are included in the article's Creative Commons licence, unless indicated otherwise in a credit line to the material. If material is not included in the article's Creative Commons licence and your intended use is not permitted by statutory regulation or exceeds the permitted use, you will need to obtain permission directly from the copyright holder.

To view a copy of this licence, visit https://creativecommons.org/licenses/by/4.0/.

## Electronic supplementary material

Supplementary material, approximately 163 KB.
