# Supplementary material for: Research progress on human infection with avian influenza H7N9
Source: Front Med. 2020 Jan 23;14(1):8–20. doi: 10.1007/s11684-020-0739-z (PMC7101792; doi:10.1007/s11684-020-0739-z)
Supplement: Supplementary file 1 — Supplementary material, approximately 163 KB. [file 11684_2020_739_MOESM1_ESM.pdf]

Supplementary Table S1 The finished H7N9 vaccine clinical trials registered in clinicalTrials.gov

Name of clinical trial	Stage	Registered number
Randomized Study of H7 Influenza Prime-Boost Regimens in Healthy Adults	I	NCT02206464
Evaluating the Safety and Immunogenicity of a H7N9 Vaccine for the Prevention of Influenza H7N9 Disease in Adults 50 to 70 Years Old	I	NCT02274545
Preliminary Study on Safety and Immunogenicity of Influenza A (H7N9) Vaccine in the Population	I	NCT03196661
Safety and Immunogenicity of GSK Biologicals' Influenza Vaccine(s) GSK3206641A and GSK3206640A Administered in Adults 18 to 64 Years of Age	I	NCT01999842
Dose-Ranging Study of Adjuvanted and Non-Adjuvanted Cell Culture-Derived, Inactivated A/H7N9Monovalent Subunit Influenza Virus Vaccine (H7N9c) in Adults 18 to <65 Years	I	NCT01928472
Evaluation of the Safety and Immunogenicity of Live Influenza A Vaccine H7N9 (6-2) AA ca Recombinant (A/Anhui/1/2013 (H7N9) x A/Ann Arbor/6/60 ca).	I	NCT01995695
The Safety And Immunogenicity Of Priming With Live Attenuated A/H7N9 Influenza Virus Vaccine Followed By Inactivated A/H7N9 Influenza Virus Vaccine With AS03 Adjuvant	I	NCT02957656
Evaluation of the Optimal Interval Between Priming With a Live Influenza A Vaccine H7N9 (6-2) AA ca Recombinant (A/Anhui/1/2013 (H7N9) x A/Ann Arbor/6/60 ca).	I	NCT02151344

Immunogenicity and Safety of Monovalent A/Anhui/1/2013 (H7N9) Virus-Like Particle (VLP) Avian Influenza Antigen (Recombinant) in Healthy Adults With and Without Adjuvant	I	NCT01897701
A Phase I Study in Healthy Adults to Assess Priming With Antigenically Mismatched Live Attenuated A/H7N3 Influenza Virus Vaccine Followed by Inactivated A/H7N9 Influenza Virus Vaccine	I	NCT03581903
A Phase I Study Priming With an Inactivated A/H7N9 Influenza Virus Vaccine With or Without MF59 Adjuvant Followed by Live Attenuated A/H7N9 Influenza Virus Vaccine	I	NCT02251288
A Study to Evaluate the Safety and Immunogenicity of H7N9 Influenza Vaccine (AT-501) in Healthy Adult Subjects	I/II	NCT02436928
Evaluate and Compare the Immunogenicity of Monovalent Inactivated Influenza A/H7N9 Virus Vaccine Administered With and Without AS03 Adjuvant and Monovalent Inactivated Influenza A/H3N2v Virus Vaccine Administered Without Adjuvant in Healthy Adults Through Standard and Systems Biology Analyses	II	NCT02921997
Safety, Reactogenicity, and Immunogenicity of an MF59-Adjuvanted, Monovalent Inactivated Influenza A/H7N9 Virus Vaccine Administered Intramuscularly at Different Intervals and Dosages	II	NCT02213354
Safety, Reactogenicity, and Immunogenicity of a Monovalent Influenza A/H7N9 Virus Vaccine Administered at Different Dosages Given With and Without AS03 and MF59 Adjuvants	II	NCT01942265

Safety, Reactogenicity, and Immunogenicity of a Single Intramuscular Dose of Inactivated Influenza A/H7N9 VaccineAfter Priming With Inactivated Influenza A/H7N7 Vaccine	II	NCT02586792
Safety, Reactogenicity, and Immunogenicity of a Monovalent Influenza A/H7N9 Virus Vaccine Administered at Different Dosages Given With and Without MF59 Adjuvant	II	NCT01938742
A Phase II Study in Healthy Adults 19 Years and Older to Assess the Safety, Reactogenicity and Immunogenicity of a Sanofi Pasteur A/H7N9Inactivated Influenza Vaccine Administered Intramuscularly With or Without AS03 Adjuvant	II	NCT03312231
A Phase II Study in Healthy Adults (19-64 Years of Age) to Assess the Safety, Reactogenicity and Immunogenicity of Sequential or Simultaneous Intramuscular Administration of an AS03-adjuvanted A/H7N9 Inactivated Influenza Vaccine With Seasonal Influenza Vaccine	II	NCT03318315

Supplementary Table S2 The ongoing H7N9 vaccine clinical trials registered in clinicalTrials.gov

Name of clinical trial	Stage	Registered number
Reactogenicity, Safety and Immunogenicity of a Live Monovalent A/17/Hong Kong/2017/75108 (H7N9) Influenza Vaccine	I	NCT03739229
A Phase II Study to Assess the Safety, Reactogenicity and Immunogenicity of a Single Dose of 2017 A/H7N9 Inactivated Influenza Vaccine (IIV) Administered Intramuscularly With or Without AS03 Adjuvant in 2013 A/H7N9 IIV Primed or A/H7 IIV Naïve Subjects	II	NCT03738241
A Phase II Study in Healthy Adults 18-64 Years Old to Assess the Safety, Reactogenicity and Immunogenicity of a Seqirus A/H7N9 Inactivated Influenza Vaccine Administered Intramuscularly With or Without MF59 (R) Adjuvant	II	NCT03682120
Immunogenicity And Safety Of An Alum-Adjuvanted Inactivated H7N9 Influenza Vaccine	I/II	NCT03369808
Immunogenicity and Safety of an Alum-adjuvanted Inactivated H7N9 Influenza Vaccine: a Randomized, Blind, Placebo-controlled, a Phase II Clinical Trial	II	NCT03755427
A Phase II Study to Assess the Safety, Reactogenicity and Immunogenicity of Different Prime-Boost Vaccination Schedules of 2013 and 2017 A/H7N9Inactivated Influenza Vaccines Administered Intramuscularly With or Without AS03 Adjuvant in Healthy Adults 19-50 Years of Age	II	NCT03589807
